# Reducing the Dietary Omega-6:Omega-3 Utilizing α-Linolenic Acid; Not a Sufficient Therapy for Attenuating High-Fat-Diet-Induced Obesity Development Nor Related Detrimental Metabolic and Adipose Tissue Inflammatory Outcomes

**DOI:** 10.1371/journal.pone.0094897

**Published:** 2014-04-14

**Authors:** Reilly T. Enos, Kandy T. Velázquez, Jamie L. McClellan, Taryn L. Cranford, Michael D. Walla, E. Angela Murphy

**Affiliations:** 1 Department of Pathology, Microbiology and Immunology, School of Medicine, University of South Carolina, Columbia, South Carolina, United States of America; 2 Department of Chemistry and Biochemistry, University of South Carolina, Columbia, South Carolina, United States of America; CRCHUM-Montreal Diabetes Research Center, Canada

## Abstract

**Aims:**

To examine the effect of manipulating the omega-6:omega-3 (1∶1, 5∶1, 10∶1, and 20∶1) utilizing only α-linolenic and linoleic acid within a clinically-relevant high-fat diet (HFD) composed of up to seven sources of fat and designed to be similar to the standard American diet (MUFA∶PUFA of 2∶1, 12% and 40% of calories from saturated and total fat, respectively) on body composition, macrophage polarization, inflammation, and metabolic dysfunction in mice.

**Methods:**

Diets were administered for 20 weeks. Body composition and metabolism (HOMA index and lipid profile) were examined monthly. GC-MS was utilized to determine the eicosapentaenoic acid (EPA):arachidonic acid (AA) and the docosahexaenoic acid (DHA):AA in AT phospholipids. Adipose tissue (AT) mRNA expression of chemokines (MCP-1, Fetuin-A, CXCL14), marker genes for M1 and M2 macrophages (CD11c and CD206, respectively) and inflammatory markers (TNF-α, IL-6, IL-1β, TLR-2, TLR-4, IL-10, GPR120) were measured along with activation of NFκB, JNK, and STAT-3. Macrophage infiltration into AT was examined using F4/80 immunohistochemistry.

**Results:**

Any therapeutic benefit produced by reducing the omega-6:omega-3 was evident only when comparing the 1∶1 to 20∶1 HFD; the 1∶1 HFD resulted in a lower TC:HDL-C and decreased AT CXCL14 gene expression and AT macrophage infiltration, which was linked to a higher EPA:AA and DHA:AA in AT phospholipids. However, despite these effects, and independent of the omega-6:omega-3, all HFDs, in general, led to similar levels of adiposity, insulin resistance, and AT inflammation.

**Conclusion:**

Reducing the omega-6:omega-3 using α-linolenic acid is not an effective therapy for attenuating obesity and type II diabetes mellitus development.

## Introduction

Given the worldwide obesity epidemic, humans are at an increased risk of developing life-threating conditions including type 2 diabetes mellitus, cardiovascular disease, and cancer [1]. Although many factors are likely responsible for the rampant development of global obesity, one of the primary culprits is a prolonged overindulgence in energy-dense, high-fat diets (HFDs).

While the absolute intake of dietary fat has undoubtedly contributed to the international expansion of obesity, the fatty-acid (FA) composition of a HFD, independent of total dietary fat, can greatly impact metabolic and inflammatory processes [2]. Recently, significant attention has surrounded the dietary ratio of omega-6 polyunsaturated fatty-acids (PUFAs) to omega-3 PUFAs (omega-6:omega-3) as it relates to chronic disease development [3], [4]. Omega-6 PUFAs are considered to be pro-inflammatory, whereas omega-3 PUFAs are considered to be anti-inflammatory, and thus presumably a decrease in the omega-6:omega-3 within a HFD would minimize the inflammatory and metabolic perturbations associated with obesity [5]. In fact, a high omega-6:omega-3 consumption has been associated with the promotion of many chronic diseases [5]. Given this, it has been recommended that the omega-6:omega-3 consumption should be 4∶1-1∶1 as is commonly seen within the populations of wild animals and our human ancestors [5], [6], [7], [8], [9]. Conversely, in the United States, the average consumption of omega-6:omega-3 is 10-20:1, a far cry from the ideal ratio [5].

To date, there is lack of scientific evidence to strongly substantiate the claim that reducing the omega-6:omega-3 would curtail obesity and its detrimental side effects as no single study has employed various clinically-relevant HFDs to examine the influence of reducing the omega-6:omega-3 on obesity development, let alone while utilizing a particular species of these PUFAs. Thus, we examined the influence of four tightly controlled, clinically-applicable, HFDs (40% of total calories from dietary fat) differing solely in the omega-6:omega-3 (1∶1, 5∶1, 10∶1, and 20∶1) using only parent-chain, plant-based, omega-6 and omega-3 FAs (linoleic (LA; C18:2) and α-linolenic acid (ALA; C18:3), respectively) on body composition, macrophage polarization, inflammation, and metabolic dysfunction in mice. We hypothesized that the higher the omega-6:omega-3 the greater the adiposity and the worse the obesity-associated outcomes.

## Methods

### Animals

Male *C57BL/6* mice were purchased from Jackson Laboratories (Bar Harbor, ME) and were cared for in the animal facility at the University of South Carolina. They were housed, 5/cage, maintained on a 12∶12-h light-dark cycle in a low stress environment (22°C, 50% humidity, low noise) and given food and water *ad libitum*. Principles of laboratory animal care were followed, and the Institutional Animal Care and Usage Committee of the University of South Carolina approved all experiments.

### Diets

At four weeks of age, mice were randomly assigned to 1 of 5 treatment diets (n = 10/group): a control diet (AIN-76A Mod) and four HFDs (1∶1, 5∶1, 10∶1, and 20∶1) (BioServ, Frenchtown, NJ) (Table 1). The percentage of calories provided by each of the three macronutrients and the ratio of monounsaturated FAs (MUFAs) to PUFAs (MUFA:PUFA) were identical for the HFDs and were designed to be similar to the standard American diet [5], [10]. The only significant difference among the HFDs was the omega-6:omega-3. None of the diets contained any long-chain omega-6 or omega-3 FAs. The control diet (AIN-76A Mod) was used in order to match the MUFA:PUFA and omega-6:omega-3 of the 20∶1 HFD.

**Table 1:** Diet composition of treatment diets.

	AIN-76A Modified	1:1 HFD	5:1 HFD	10:1 HFD	20:1 HFD
Ingredient (g/kg)					
Casein	200	165	165	165	165
DL Methionine	3	3	3	3	3
Lard	0	100	100	100	100
Coconut Oil	0	10.2	7.3	6.8	7.4
Corn Oil	15.6	.9	.5	22.5	50.1
Soybean Oil	3.6	1	41.1	28.1	3.8
Olive Oil	30.9	45	40	43.7	41.7
Flaxseed Oil	-	38.5	4.9	1	-
Canola Oil	-	7.4	9.2	1	-
Corn Starch	80	50	50	50	50
Maltodextrin	100	100	100	100	100
Sucrose	469.5	381.9	381.9	381.9	381.9
Cellulose	50	50	50	50	50
Vitamin Mix (AIN-76A)	10	10	10	10	10
Mineral Mix (AIN-76A)	35	35	35	35	35
Choline Bitartrate	2	2	2	2	2
Energy (kcal/g)	3.77	4.572	4.572	4.572	4.572
Energy (% kcal)					
Carbohydrate	68.7	47	47	47	47
Fat	12.2	40	40	40	40
Protein	19.1	13	13	13	13
Fatty Acid Profile (g/kg)					
Caprylic Acid (C8:0)	0	0.8	0.5	0.5	.6
Capric Acid (C10:0)	0	0.7	0.5	0.5	.5
Lauric Acid (C12:0)	0	4.8	3.5	3.2	3.5
Myristic Acid (C14:0)	.004	3.1	2.5	2.4	2.6
Palmitic Acid (C16:0)	5.5	32.2	33.9	34.7	34.8
Palmitoleic Acid (C16:1)	.4	3.3	3.2	3.3	3.3
Stearic Acid (C18:0)	1.05	16.2	16.7	16.3	15.6
Oleic Acid (C18:1)	27.1	85.9	86.1	86	85.9
Linoleic Acid (C18:2)	13.2	22.6	37.9	41.3	43.2
α-Linolenic Acid (C18:3)	.66	22.6	7.6	4.13	2.2
% of Total Calories from SFAs	1.7%	12%	12%	12%	12%
% of Total Calories from MCFAs (C6:0-C12:0)	-	1.3%	.9%	.9%	1%
% of Total Calories from LCSFAs (C14:0-C20:0)	1.7%	10.7%	11.1%	11.1%	11%
% of Total Calories from USFAs	10.5%	28%	28%	28%	28%
% of Total Calories from MUFAs	7%	18.6%	18.6%	18.6%	18.6%
% of Total Calories from PUFAs	3.5%	9.4%	9.4%	9.4%	9.4%
% of Total Calories from n-3 FAs	.16%	4.7%	1.6%	.86%	.46%
% of Total Calories from n-6 FAs	3.2%	4.7%	7.8%	8.6%	9%
Cholesterol (mg/kg)	0	95	95	95	95
Ratio: MUFA:PUFA	2:1	2:1	2:1	2:1	2:1
Ratio: n-6:n-3 FA	20:1	1:1	5:1	10:1	20:1

SFAs, Saturated Fatty-Acids; MCSFAs, Medium-Chain Saturated Fatty Acids; LCSFAs, Long-Chain Saturated Fatty Acids; USFAs, Unsaturated Fatty Acids; MUFAs, Monounsaturated Fatty Acids; PUFAs, Polyunsaturated Fatty Acids.

### Body weights, food intake, and body composition

Body weight and food intake were monitored weekly. Body composition was assessed every four weeks (age 4, 8, 12, 16, 20, and 24 weeks) via dual-energy x-ray absorptiometry (DEXA) (Lunar PIXImus, Madison, WI).

### Metabolism

Plasma was assessed for fasting (5 hr) concentrations of glucose, insulin, total cholesterol (TC), HDL-C, LDL-C, and triglycerides at age 8, 12, 16, 20, and 24 weeks as previously described [2]. Insulin resistance was estimated by HOMA index as follows: insulin resistance index  =  fasting insulin (µU/ml) x fasting glucose (mmol/l)/22.5 [11].

### Tissue collection

At 24 weeks of age, mice were sacrificed for tissue collection. Tissues were removed, weighed, and immediately snap-frozen in liquid nitrogen and stored at −80°C or fixed in 10% formalin until analysis.

### Adipocyte size and F4/80 immunohistochemistry

The mean adipocyte size determination as well as H&E and F4/80 staining was performed in epididymal AT as previously described [2].

### Western blots

Inflammatory signaling pathways were measured in epididymal fat via western blot analysis using primary antibodies for phosphorylated (Ser536) and total NFκB p65, phosphorylated (Thr183/Tyr185) and total JNK, and phosphorylated (Tyr705) and total STAT3 (Cell Signaling, Danvers, MA) [2].

### Gene expression

Quantification of epididymal AT gene expression for chemokines (MCP-1, Fetuin-A, CXCL14), macrophage (F4/80, CD11c, CD206) and inflammatory markers (TNF-α, IL-6, IL-1β, TLR-2, TLR-4, IL-10) and GPR-120 (Applied Biosystems, Foster City, CA) were performed as previously described [2].

### EPA:AA and DHA:AA in AT phospholipids

AT samples were homogenized in a 2∶1 (v/v) chloroform:methanol solution containing 100 mg/liter of butylated hydroxyl toluene in order to minimize autoxidation of PUFAs. Lipids were subsequently isolated using the folch extraction method [12]. The solution containing the lipids was dried under nitrogen gas and re-solubilized in chloroform. The lipid solution was then added to silica Sep-Pak cartridges (Waters Associated, Milford, MA). Neutral lipids, glycolipids, and phospholipids were eluted with chloroform, acetone, and methanol, respectively [13]. The phospholipid fraction was dried under nitrogen gas prior to the addition of methyl acetate in order to form fatty-acid methyl esters. After a 50°C incubation overnight, the phospholipid solution was dried under nitrogen gas, re-solubilized in chloroform, and was injected into the GC-MS in order to determine PUFA AT phospholipid composition.

GC-MS analysis was performed on a HP-5890 gas chromatograph interfaced to a VG-70S magnetic sector mass spectrometer. The column used was a 30 meter by 0.25 mm ID Restek FameWax (Bellefonte, PA). The oven temperature was programmed from 70°C to 200°C at 10°C/min and then to 250°C at 4°C/min where it was held for 8 min. The mass spectrometer was scanned from 60 to 390 Da. The retention times were compared to a marine oil FAME MIX standard (Restek).

### Statistical analyses

All data were analyzed using commercial software (SigmaStat, SPSS, Chicago, IL). Body weight, body composition outcomes, and metabolic outcomes were analyzed using a repeated measures two-way ANOVA. All other data were analyzed using a one-way ANOVA. Student-Newman-Keuls test was used for all post-hoc analyses. For the AA:EPA, a two-tailed student's t-test was used to compare the 1∶1 and 5∶1 HFD groups as EPA content was undetectable in all other groups. Statistical significance was set with an alpha value of P≤0.05. Data are presented as mean (±SEM).

## Results

### The EPA (C20:5 n-3):AA and DHA (C20:6 n-3):AA (C20:4 n-6) in AT phospholipids is greatly impacted by the omega-6:omega-3

**Figure 1:**
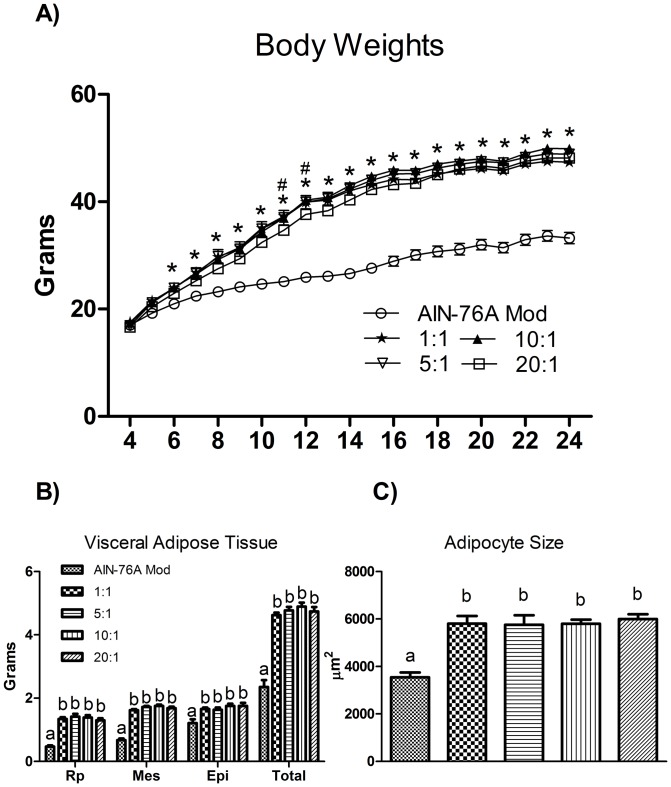
Representative (A) GC-MS chromatograms for each diet showing peaks representing AA, EPA, and DHA in AT phospholipids. (B) EPA:AA and (C) DHA:AA in AT phospholipids (n = 10). Diets not sharing a common letter differ significantly from one another (P≤.05). ND  =  Not Detected.

EPA content was only detected in the 1∶1 and 5∶1 HFD groups, with the 1∶1 group exhibiting a significantly higher EPA:AA than the 5∶1 group (P≤.05) (Figure 1A, 1B).

The DHA:AA in AT phospholipids was influenced by the dietary omega-6:omega-3 in a dose-dependent manner; all HFDs resulted in a significantly different DHA:AA (P≤.05), with the 1∶1 group resulting in the highest DHA:AA followed by the 5∶1, 10∶1, and 20∶1 groups, respectively (Figure 1A, 1C). The only HFD group not different from the control group was the 20∶1 HFD.

### No difference in adiposity among HFDs

**Figure 2:**
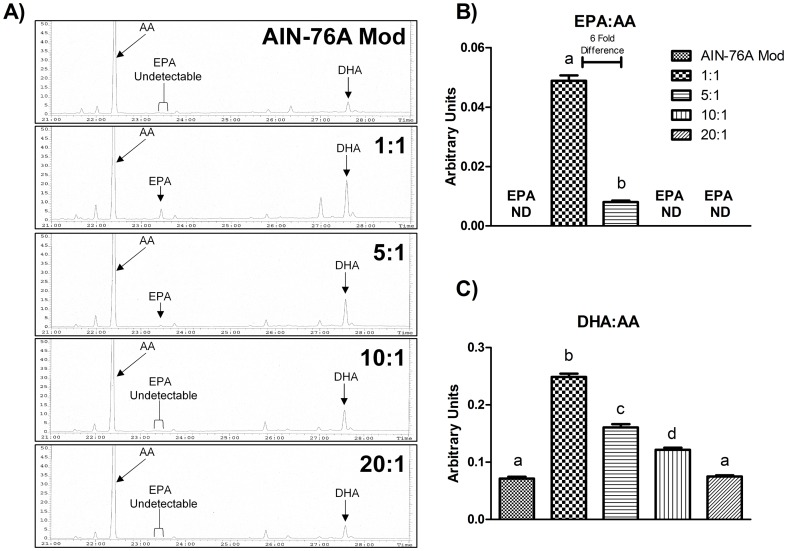
Influence of diets on (A) weekly mean body weight, (B) fat pad weights (retroperitoneal, mesentery, epididymal), and (C) adipocyte size at sacrifice (n = 10). Diets not sharing a common letter differ significantly from one another (P≤.05). ^*^Significantly different from AIN-76A Mod (ages 6–24 weeks: all HFDs) ^#^Significantly different from 20:1 (11 weeks of age: 10:1, 12 weeks of age: 5:1) (P≤.05).

Beginning at 6 and 7 weeks of age, the 1∶1, 5∶1, 10∶1 HFD-fed mice, and the 20∶1 HFD-fed mice, respectively, had significantly heavier (P≤.05) body weights compared to control-fed mice (Figure 2). In general, there were no differences in body weight among any of the HFD groups except for at 11 (10∶1>20∶1) and 12 (5∶1>20∶1) weeks of age (P≤.05).

For the fat-pad depots and adipocyte size, the HFD groups exhibited enhanced visceral fat mass and larger adipocytes compared to control-fed mice only (P≤.05).

Analysis of body composition revealed a significant difference among groups (P≤.05) (Table 2). Specifically, starting at 8 weeks of age, all HFD groups had a greater fat mass, and body fat percentage compared to control-fed mice (P≤.05). The only differences in fat mass accrual among the HFD groups were at age 8 (5∶1>20∶1) and 12 (1∶1 and 5∶1>20∶1) weeks and in body fat percentage at age 8 (1∶1 and 5∶1>10∶1 and 20∶1) and 12 (5∶1>20∶1) weeks (P≤.05). In general, lean mass increased over time for all groups, and by 8 weeks of age, the HFD-fed mice exhibited greater lean mass compared to control-diet-fed mice (P≤.05).

**Table 2:** Body composition of mice assessed at baseline (4 weeks of age) and incrementally (age 8, 12, 16, 20, and 24 weeks) throughout the course of the study (n = 10).

Fat Mass (Grams)						
Diet	Week 4	Week 8	Week 12	Week 16	Week 20	Week 24
AIN-76A Mod	1.6±.10^a^	4.1±.18^b@^	4.7±.24^b@^	6.6±.50^c@^	8.0±.65^d@^	9.3±.74^e@^
1:1	1.6±.08^a^	7.5±.59^b#∧^	14.3±.42^c#^	17.3±.33^d#^	18.9±.49^e#^	19.9±.43^f#^
5:1	1.6±.10^a^	7.9±.78^b#^	14.7±.70^c#^	18.2±.45^d#^	20.0±.49^e#^	21.4±.62^f#^
10:1	1.7±.09^a^	6.5±.53^b#∧^	13.4±.73^c#∧^	18.0±.46^d#^	19.6±.71^e#^	20.3±.59^e#^
20:1	1.6±.11^a^	5.9±.43^b∧^	12.6±.69^c#∧^	17.1±.76^d#^	19.5±.54^e#^	19.8±.54^e#^
**Lean Mass (Grams)**						
AIN-76A Mod	13.0±.43^a^	16.5±.34^b@^	18.7±.44^c@^	19.4±.42^c@^	20.7±.52^d@^	20.9±.49^d@^
1:1	13.5±.40^a^	18.9±.61^b#^	21.6±.41^c#^	23.0±.42^d#^	23.4±.39^d#^	23.5±.27^d#^
5:1	12.7±.45^a^	18.5±.48^b#^	21.4±.62^c#^	23.0±.54^d#∧^	23.6±.55^d#^	23.4±.52^d#^
10:1	13.2±.52^a^	19.4±.22^b#^	22.7±.37^c#^	24.2±.39^d∧^	24.7±.44^de#^	25.6±.56^e∧^
20:1	12.5±.45^a^	18.6±.37^b#^	21.2±.40^c#^	22.5±.40^d#^	23.6±.41^e#^	24.6±.32^f#∧^
**Body Fat %**						
AIN-76A Mod	11.0±.38^a^	19.9±.75^b@^	20.0±.67^b@^	25.1±1.2^c@^	27.5±1.6^d@^	30.3±1.6^e@^
1:1	10.6±.32^a^	28.2±1.4^b#^	39.8±.83^c#∧^	43.0±.73^d#^	44.6±.83^de#^	45.8±.50^e#^
5:1	10.9±.36^a^	29.4±1.7^b#^	40.6±1.0^c#^	44.3±.90^d#^	46.0±.69^de#^	47.7±1.0^e#^
10:1	11.0±.35^a^	24.9±1.6^b∧^	37.0±1.4^c#∧^	42.6±.82^d#^	44.1±1.2^d#^	44.2±1.1^d#^
20:1	10.9±.57^a^	23.9±1.5^b∧^	37.0±1.5^c∧^	43.0±1.4^d#^	45.2±1.0^e#^	44.6±.93^e#^

Values not sharing a common letter differ significantly over time within the given diet (P≤.05). Values not sharing a common symbol differ significantly among diet within the given week (P≤.05).

It was not possible to calculate individual food intake as mice were housed 5/cage. However, in general, we did not observe any differences, among the HFD-fed mice, in weekly food intake (food consumed by mice in each cage/number of mice in cage) over the course of the study.

### All HFDs lead to similar levels of inflammatory markers despite the fact that the 1∶1 reduces AT macrophage infiltration

**Figure 3:**
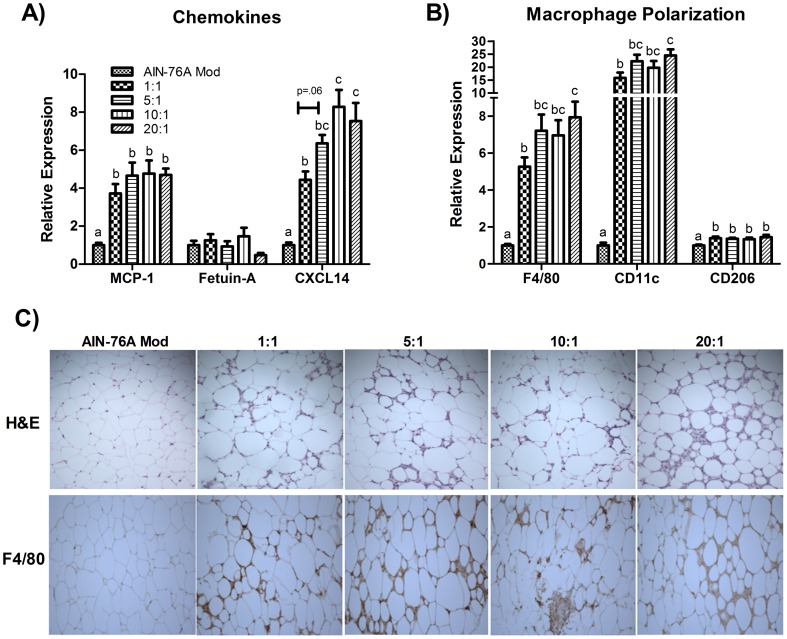
Epididymal AT gene expression of (A) chemokines and (B) macrophage markers (n = 10). Diets not sharing a common letter differ significantly from one another (P≤.05). Representative images of (C) H&E and F4/80 staining of epididymal AT (20x).

All HFDs increased the gene expression of the chemokines MCP-1 and CXCL14 compared to the control diet, whereas there were no differences in Fetuin-A mRNA across diets (P≤.05) (Figure 3A). When comparing among the HFD groups, CXCL14 mRNA was found to be significantly upregulated (P≤.05) in the 10∶1 and 20∶1 HFD and trending to be upregulated in the 5∶1 group (p = .06) compared to the 1∶1 group.

Regarding macrophage polarization, each HFD upregulated the gene expression of F4/80, CD11c, and CD206 in the AT compared to the control diet (P≤.05) (Figure 3B). However, the 1∶1 group displayed significantly less mRNA expression of F4/80 and CD11c as compared to the 20∶1 group. These findings were confirmed via H&E and immunohistochemistry staining of F4/80 (Figure 3C).

**Figure 4:**
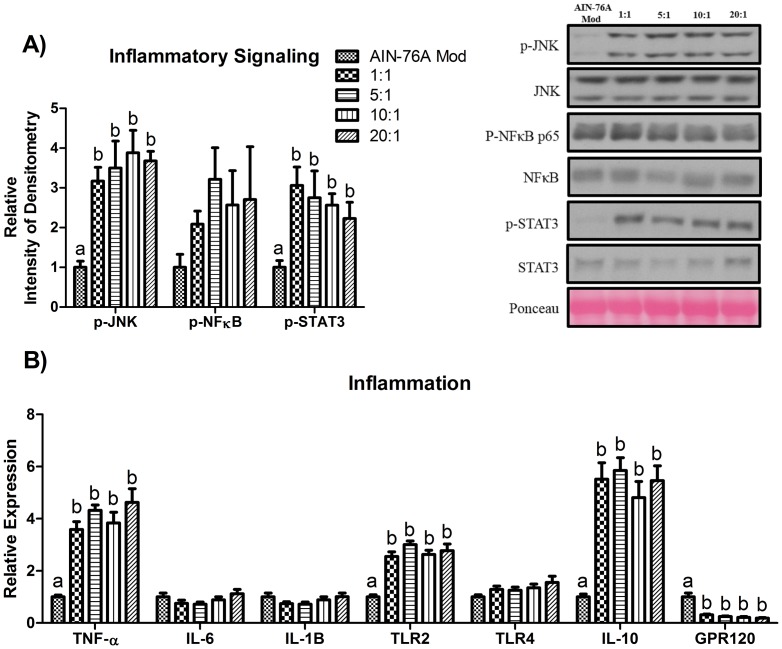
Representative epididymal AT western blots of (A) phosphorylated (Thr183/Tyr185) and total JNK, phosphorylated p65 (Ser536) and total NFκB, and phosphorylated (Tyr705) and total STAT3. Epididymal AT gene expression of (B) inflammatory markers (n = 10). Diets not sharing a common letter differ significantly from one another (P≤.05).

Despite the fact that the 1∶1 diet reduced macrophage infiltration into AT, this seemed to have no effect on the inflammatory markers measured; all HFDs equally increased the AT protein concentration of activated JNK and STAT3 (P≤.05) (Figure 4A) and mRNA expression of genes linked to pro-inflammatory processes: TNF-α, TLR2, as well as the anti-inflammatory cytokine, IL-10 (Figure 4B). Additionally, manipulating the omega-6:omega-3 did not modulate the expression of the omega-3 and long-chain FA receptor, GPR120, as all HFDs down-regulated the expression of GPR120 relative to the control diet (P≤.05) (Figure 4B). Regarding the other inflammatory markers examined, no differences were detected in the protein concentration of p-NFκB (Figure 4A) nor in the gene expression of IL-6, IL-1β, and TLR4 (Figures 4B).

### Reducing the omega-6:omega-3 has no effect at blunting the development of insulin resistance

Beginning at 12 weeks of age, all HFD groups had higher fasting blood glucose (FBG) concentrations compared to the control group (P≤.05), however, at week 20, there were no differences in FBG across groups, except for the 5∶1 group, which exhibited a significantly lower FBG compared to all other HFD-groups (P≤.05) (Table 3). At 24 weeks of age, all HFD groups featured FBG levels greater than the control group, save for the 10∶1 group – which also displayed FBG levels significantly lower than the 5∶1 group (P≤.05). With regard to fasting plasma insulin, all HFD groups displayed higher insulin levels, and a greater HOMA index compared to the control group starting at week 12 weeks of age (P≤.05).

**Table 3:** Fasting metabolic panel assessed incrementally (age 8, 12, 16, 20, and 24 weeks) throughout the course of the study (n = 10).

Table 3. *Metabolism*					
**Glucose (mmol/l)**					
**Diet**	**Week 8**	**Week 12**	**Week 16**	**Week 20**	**Week 24**
AIN-76A Mod	8.9±0.6^a^	8.0±0.4^a@^	9.9±0.5^bc@^	9.9±0.8^bc@#^	8.8±0.4^ab@^
1:1	8.9±0.5^a^	11.3±0.7^b#^	13.0±0.7^c#^	11.2±0.7^b@^	10.6±0.5^b#∧^
5:1	10.1±0.5^ac^	12.5±0.5^b#^	11.9±0.6^b#^	9.3±0.3^a#^	11.4±0.6^bc#^
10:1	9.6±0.3^a^	10.9±0.3^b#^	11.8±0.3^b#^	11.1±0.6^b@^	9.2±0.3^a@∧^
20:1	9.0±0.2^a^	11.4±0.3^b#^	11.8±0.4^b#^	11.3±0.4^b@^	10.7±0.4^b#∧^
**Insulin (**µ**U/mL)**					
AIN-76A Mod	24.1±2.2	24.2±1.8^@^	32.1±2.9^@^	34.5±4.8^@^	31.4±3.3^@^
1:1	41.6±4.8^a^	113±7.8^b#^	138±13.6^bc#^	122±8.4^b#^	159±16.1^c#^
5:1	36.7±5.3^a^	109±13.3^b#^	131±9.0^c#^	138±10.9^c#^	153±10.9^c#^
10:1	34.5±4.0^a^	102±11.4^b#^	145±9.5^c#^	141±13.8^c#^	161±23.5^c#^
20:1	36.4±3.9^a^	87.3±10.6^b#^	121±10.6^c#^	144±11.2^c#^	142±10.1^c#^
**HOMA (unit)**					
AIN-76A Mod	9.7±1.1	8.8±1.0^@^	14.0±1.6^@^	14.9±2.8^@^	13.0±1.8^@^
1:1	16.3.±2.7^a^	56.1±6.3^b#^	79.2±8.5^c#^	61.2±6.5^b#^	77.3±10.5^c#^
5:1	16.8±3.3^a^	60.3±7.2^b#^	74.1±5.7^c#^	59.0±4.9^b#^	74.2±4.2^c#^
10:1	14.7±1.4^a^	52.3±6.9^b#^	76.9±5.7^c#^	69.4±8.6^cd#^	61.2±9.0^bcd#^
20:1	14.6±1.6^a^	44.6±5.6^b#^	63.2±6.1^c#^	72.3±5.9^c#^	63.5±2.1^c#^
**Total Cholesterol (mmol/l)**					
AIN-76A Mod	3.91±0.11	3.80±0.12^@^	3.91±0.16^@^	3.93±0.20^@^	4.08±0.17^@^
1:1	4.05±0.18^a^	4.43±0.13^b#^	4.76±0.16^c#^	5.04±0.12^d#^	5.19±0.16^d#^
5:1	4.35±0.12^a^	4.56±0.14^b#^	4.89±0.18^c#^	5.26±0.18^d#^	5.49±0.17^e#^
10:1	4.18±0.14^a^	4.63±0.07^b#^	4.91±0.10^c#^	4.79±0.22^c#^	5.30±0.16^d#^
20:1	4.30±0.11^a^	4.58±0.12^b#^	4.82±0.15^c#^	5.13±0.17^d#^	5.52±0.18^e#^
**LDL-C (mmol/l)**					
AIN-76A Mod	1.10±0.04^a^	0.98±0.04^a@^	1.05±0.04^a@^	1.07±0.08^a@^	1.31±0.06^b@^
1:1	1.29±0.09^a^	1.93±0.07^b#^	2.02±0.05^b#^	2.17±0.09^c#^	2.50±0.12^d∧^
5:1	1.42±0.05^a^	1.82±0.07^b#^	2.07±0.09^c#^	2.03±0.10^c#^	2.40±0.12^d∧$^
10:1	1.24±0.09^a^	1.56±0.08^b#^	1.89±0.10^c#^	1.90±0.13^c#^	2.07±0.12^c#^
20:1	1.43±0.10^a^	1.74±0.10^b#^	1.90±0.07^c#^	2.02±0.11^cd#^	2.18±0.13^d#$^
**HDL-C (mmol/l)**					
AIN-76A Mod	1.33±0.05^a@^	1.25±0.03^a^	1.24±0.03^a@^	1.10±0.03^b@^	0.97±0.02^c@^
1:1	1.63±0.04^a∧^	1.22±0.03^b^	1.35±0.03^c#^	1.36±0.03^c#^	1.20±0.02^b#^
5:1	1.51±0.06^a#^	1.29±0.05^b^	1.22±0.02^bc@#^	1.24±0.03^bc#∧^	1.14±0.03^c#^
10:1	1.49±0.06^a#^	1.31±0.04^bd^	1.12±0.03^c@∧^	1.26±0.04^d#∧^	1.11±0.06^c#^
20:1	1.47±0.03^a#^	1.32±0.04^b^	1.10±0.02^c∧^	1.18±0.03^c@∧^	1.12±0.03^c#^
**TC:HDL-C**					
AIN-76A Mod	3.0±0.2	3.0±0.1	3.2±0.2^@^	3.5±0.2^@^	4.0±0.2^@^
1:1	2.6±0.2	3.7±.02	3.4±0.1^@#^	3.8±0.2^@#^	4.3±0.2^@^
5:1	2.9±0.2	3.6±0.2	4.0±0.2^#∧^	4.2±0.3^@#^	4.9±0.2^#^
10:1	2.9±0.2	3.5±0.2	4.5±0.1^∧^	3.8±0.3^@#^	4.7±0.3^@#^
20:1	3.0±0.1	3.5±0.2	4.4±0.2^∧^	4.4±0.2^#^	5.0±0.2^#^
**Triglycerides (mmol/l)**					
AIN-76A Mod	0.91±0.03	0.89±.03	0.94±0.02	0.93±0.02	0.91±0.03
1:1	0.92±0.05	0.94±.03	0.98±0.04	0.97±0.04	0.96±0.03
5:1	0.91±0.03	0.92±0.03	0.95±0.02	0.92±0.03	0.92±0.04
10:1	0.90±0.03	0.91±0.04	0.98±0.02	0.92±0.3	0.90±0.03
20:1	0.88±0.01	0.90±0.04	0.97±0.02	0.91±0.02	0.94±0.04

Values not sharing a common letter differ significantly over time within the group (P≤.05). Values not sharing a common symbol differ significantly among groups within the given week (P≤.05).

### Plasma lipid profile is influenced by omega-6:omega-3

All HFD groups increased total cholesterol and LDL-C levels compared to the control group starting at 12 weeks of age (P≤.05) (Table 3). However, only a difference in LDL-C was detected among the HFD groups, which occurred solely at week 24; the 10∶1 group presented a significantly lower LDL-C concentration than both the 1∶1 and 5∶1 groups, as did the 20∶1 group when compared to the 1∶1 group (P≤.05).

With regard to HDL-C, consumption of each of the HFDs resulted in elevated HDL-C levels compared to control-diet consumption starting at 8 weeks of age (P≤.05). Further, among HFD groups, the 1∶1 group had the greatest HDL-C (P≤.05). No differences in HDL-C were detected among any of the groups at 12 weeks of age. However, at 16 weeks of age, both the 1∶1 and 5∶1 diets produced significantly higher HDL-C levels than the 20∶1 diet and also the 10∶1 and control diets in the case of the 1∶1 group. The 20∶1 group, on the other hand, resulted in significantly lower HDL-C than the control group (P≤.05). At 20 weeks of age, all HFDs, except for the 20∶1 diet, resulted in a higher HDL-C concentration compared to the control diet (P≤.05). Similar to 16 week old mice, consumption of the 1∶1 diet led to a greater concentration of HDL-C more so than the 20∶1 diet (P≤.05). Finally, at 24 weeks of age, all HFD groups had higher levels of HDL-C compared to the control group (P≤.05), but there were no differences among them.

Differences in the TC:HDL-C were not detected until 16 weeks of age; the 5∶1, 10∶1, and 20∶1 groups had a higher TC:HDL-C compared to the control group (P≤.05), and the 1∶1 group displayed a significantly lower TC:HDL-C relative to the 10∶1 and 20∶1 groups (P≤.05). At 20 weeks of age, although there were no differences in TC:HDL-C among the HFD groups, only the 20∶1 group had a significantly elevated TC:HDL-C compared to the control group (P≤.05). At the endpoint of the study, both the consumption of the 5∶1 and 20∶1 diets led to a higher TC:HDL-C compared to the control and 1∶1 diets (P≤.05).

No differences were detected in triglyceride levels at any time point among any of the groups throughout the course of the study.

## Discussion

Evidence indicates that omega-3 FAs have the potential to offset the detrimental effects of a HFD, including reduced adiposity and inflammation, and improve insulin sensitivity [14], [15]. However, the majority of such studies have used one of the two long-chain PUFAs, EPA or DHA, alone, or in combination. Minimal research on the prospective benefits of the short-chain omega-3 FA, ALA, on obesity development has been performed. This is surprising given that ALA is the most prominent dietary PUFA consumed [16] and is a common ingredient that fortifies many commercially-available foods [17]. Further, although the public is constantly bombarded with claims to reduce the dietary omega-6:omega-3 in order to improve overall health, there is a lack of properly controlled investigations to support such a claim.

Given this, we sought to examine the impact of reducing the omega-6:omega-3 ratio utilizing only ALA in order to be able to draw concise conclusions about the possible health benefits of the short-chain omega-3 FA on a variety of cellular and metabolic perturbations stemming from obesity. Our manipulation of the omega-6:omega-3 was grounded in a diet that contained 40% of total calories from fat, 12% calories from saturated fat and a MUFA:PUFA of 2∶1. It utilized up to seven different sources of fat so that the total consumption of omega-3 PUFAs in the diet fell within a clinically-appropriate dose (4.7%, 1.6%, .86%, and .46% of total calories from omega-3 PUFAs for the 1∶1, 5∶1, 10∶1, and 20∶1 diets, respectively; it has been reported that the typical American's diet is composed of up to .2-.7% of total calories from omega-3 PUFAs [16]), and the only FA ratio which changed in the diet was the omega-6:omega-3 [10].

Although there are several means by which omega-3 FAs are thought to elicit their therapeutic effects on attenuating obesity development, the three primary mechanisms are as follows: 1) enhanced ability of omega-3 FAs to be oxidized rather than stored [18], 2) modulation of the phospholipid composition of the cellular membrane influencing the biosynthesis of inflammatory eicosanoids [19], and 3) ability to bind GPR120 and inhibit pro-inflammatory pathways [20].

Of all unsaturated FAs, ALA is reported to be one of the most efficiently oxidized [18], [21]. Thus, we hypothesized that decreasing omega-6:omega-3 would favor a reduction in adiposity resulting from the ability to rapidly metabolize an increased consumption of ALA. However, this did not appear to be the case; after 20 weeks of HFD consumption, there was no difference in adipocyte size, total visceral AT mass, or body composition between any of the HFD groups. This may be due to the fact that LA is also an efficiently metabolized FA and any difference in the proficiency to metabolize ALA vs. LA or the absolute discrepancy in the consumption of ALA vs. LA was not sufficient enough to elicit a difference in adiposity. However, metabolic flux experiments would need to be performed in order to conclusively corroborate these findings.

Arguably, the most potent means by which omega-3 FAs elicit favorable health outcomes is through their ability to influence lipid metabolite production via modulation of cellular membrane composition. AA, the long-chain omega-6 FA, is present in great abundance in the cellular membrane – much more so than the long-chain omega-3 FAs EPA and DHA. AA serves as a precursor for the production of pro-inflammatory metabolites, including prostaglandin E_2_ (PGE_2_), whereas EPA and DHA serve as substrates for anti-inflammatory lipid metabolites [5]. LA and ALA are of importance as they serve as the substrates for elongation into AA and EPA and DHA, respectively [5]. Because both LA and ALA compete for the same enzymes for conversion into their longer-chain counterparts, the omega-6:omega-3 is of importance as an excess consumption of LA or ALA results in an increased conversion of the more abundant short-chain FA into the species-similar longer-chain PUFA. Thus, consumption of a diet with a high omega-6:omega-3 would theoretically lead to greater incorporation of AA and a decreased inclusion of EPA and DHA into the cellular membrane leading to the production of more pro-inflammatory markers compared to a diet with a lower omega-6:omega-3. However, the ability for ALA to be converted to EPA and DHA is limited as previous studies have shown that <10% and <1% of ALA is converted to EPA and DHA, respectively [18]. Given the fact that incorporation of omega-3 FAs into the cellular membrane is an integral part of the omega-3 FAs' ability to influence inflammatory processes, we examined the EPA:AA and DHA:AA in AT phospholipids. Regarding the EPA:AA, EPA was only detectable in the 1∶1 and 5∶1 groups, with the 1∶1 group exhibiting ≈ a 6-fold increase in the EPA:AA compared to the 5∶1 group. In terms of the DHA:AA, we found a dose-dependent effect – the lower the omega-6:omega-3, the greater the DHA:AA. These findings suggest that a dietary 10∶1 and 20∶1 omega-6:omega-3 utilizing ALA is not sufficient enough to allow for the incorporation of EPA into AT phospholipids. However, it seems that regardless of the dietary omega-6:omega-3, the ratio does influence the DHA:AA in a dose-dependent manner.

We next examined how changes in the AT phospholipid composition influenced AT macrophage polarization, inflammatory markers, and metabolic processes. We first investigated the gene expression of three chemokines shown to play a role in AT macrophage infiltration: MCP-1, Fetuin-A, and CXCL14 [22], [23], [24]. Independent of the omega-6:omega-3 and the percentage of total fat in the diet, all HFDs upregulated MCP-1 and CXCL14 gene expression compared to the control diet. However, CXCL14 mRNA was found to be lower in the 1∶1 HFD compared to all other HFDs. Regarding macrophage polarization, all HFDs upregulated the gene expression of the general macrophage marker, F4/80, and the M1 (CD11c) and M2 (CD206) pro- and anti-inflammatory macrophage markers, respectively. However, in both the F4/80 and CD11c analyses, consumption of the 1∶1 HFD resulted in significantly less mRNA of these markers than the 20∶1 HFD. These findings should be substantiated using fluorescence activated cell sorting methodology to firmly establish the influence of manipulating the omega-6:omega-3 with ALA on macrophage infiltration and polarization during HFD-induced obesity. Our analysis of inflammatory signaling showed that all HFDs increased the activation of JNK and STAT3, but failed to increase phosphorylation of NFκB. Similarly, regarding expression of inflammatory markers, all HFDs upregulated the pro-inflammatory markers, TNF-α and TLR2, as well as the anti-inflammatory cytokine, IL-10, likely as a compensatory mechanism for the increased inflammation. With regard to metabolic perturbations, manipulating the omega-6:omega-3 had no effect at attenuating insulin resistance nor hypercholesterolemia. However, the 1∶1 HFD did lead to a lower TC:HDL-C compared to the 5∶1 and 20∶1 HFDs, but also significantly increased LDL-C compared to all other HFDs.

Taken together, our findings regarding AT phospholipid composition and the outcomes related to macrophage polarization, inflammation, and insulin resistance suggest that changes in phospholipid composition leading to an increase in the EPA:AA and/or DHA:AA may lead to reduced macrophage infiltration, possibly via regulation of CXCL14, but does not necessarily reduce AT inflammation, as assessed by the inflammatory markers measured, nor hinder the development of insulin resistance. CXCL14 has been shown to be an efficient chemokine for PGE_2_-treated monocytes [25]. As previously mentioned, PGE_2_ is a well-known metabolite of AA metabolism. Thus it is likely that the higher EPA:AA and DHA:AA induced by the 1∶1 HFD - in particular, the EPA:AA, as this was the one diet to dramatically increase this ratio compared to all other diets - led to a diminution in the production of PGE_2_, subsequent downregulation of CXCL14, and ultimately a reduction in AT macrophage infiltration. However, despite the decreased infiltration of macrophages, there were no differences with respect to the inflammatory markers measured or insulin resistance. This was surprising given that macrophages, M1 macrophages, in particular, serve as a potent source of AT inflammation [26]. Nonetheless, adipocytes are also strong producers of inflammation and may have contributed to the inflammatory response.

It is important to note that macrophage and inflammatory markers were measured at only one time point. Hence, a full understanding of the interaction between macrophages and inflammatory outcomes would be better understood if multiple sacrifice time-points had been performed. Further, it should be noted that, although several markers of AT inflammation were assessed in this investigation, it was not feasible to perform analyses on all inflammatory mediators. Thus, other inflammatory markers may have been differently affected as a result of the dietary manipulation of the omega-6:omega-3 which were not directly examined in this study. Further, insulin resistance was assessed by the HOMA Index. A limitation of the HOMA Index is that it is insensitive to ascertaining tissue-specific insulin resistance. Thus, it is possible that there may have been differences in tissue-specific insulin sensitivity resulting from the omega-6:omega-3 manipulation that was not detected by the HOMA Index.

Omega-3 FAs have recently been shown to regulate inflammatory processes via GPR120 stimulation [20]. In addition to mediating omega-3 FA's anti-inflammatory effects, GPR120 also serves as a regulator of glucose metabolism [20] and adipogenesis [27]. Dysfunction of GPR120 is strongly associated with obesity and insulin resistance and its expression is increased in adipose tissue of HFD-fed mice [20], [27], [28]. We found that manipulating the omega-6:omega-3 had no influence on GPR120 AT gene expression. Additionally, contrary to the findings of others, we found GPR120 mRNA to be down-regulated in all HFD groups compared to the control mice. This discrepancy between studies may possibly be explained by the difference in HFDs utilized to invoke HFD-induced obesity as other studies showing upregulation of adipose tissue GPR120 gene expression in obese mice have utilized diets composed of considerably more fat (60% of total calories from fat) and long-chain FAs, which serve as natural ligands of GPR120 [29]. Nonetheless, it is evident that further investigations need to be performed in order to better understand the role of GPRs with regard to inflammatory and metabolic processes in the settings of obesity.

We would like to point out that the results of this study do not suggest that ALA does not hold important physiological functions or therapeutic properties. In fact, ALA is an essential FA required for the endogenous synthesis of EPA and DHA. Further, studies have shown that ALA may possess cardio-protective and anti-inflammatory qualities [30]. It is likely that ALA exhibits such qualities most distinctly when it is consumed in place of long-chain saturated FAs, largely characterized as pro-inflammatory.

In conclusion, this study suggests that reducing the omega-6:omega-3 solely with ALA is not an effective therapy for mitigating obesity nor type II diabetes mellitus development. It is likely that reducing the omega-6:omega-3 utilizing EPA and DHA may be a more effective strategy for attenuating obesity and related health complications, as EPA and DHA, in addition to being potent activators of GPR120, have the capability to be directly stored in phospholipids rather than having to compete for enzymes to be synthesized and then incorporated into the cellular membrane. However, an appropriately designed study needs to be conducted in order to substantiate such a hypothesis.
