# Correction: Reducing the Dietary Omega-6:Omega-3 Utilizing α-Linolenic Acid; Not a Sufficient Therapy for Attenuating High-Fat-Diet-Induced Obesity Development Nor Related Detrimental Metabolic and Adipose Tissue Inflammatory Outcomes

**DOI:** 10.1371/journal.pone.0103378

**Published:** 2014-07-17

**Authors:** The PLOS ONE Staff

The images for Figures 1 and 2 are incorrectly switched. The image that appears as Figure 1 should be Figure 2, and the image that appears as Figure 2 should be Figure 1. The figure legends appear in the correct order. The figures in their correct order can be viewed below.

**Figure 1:**
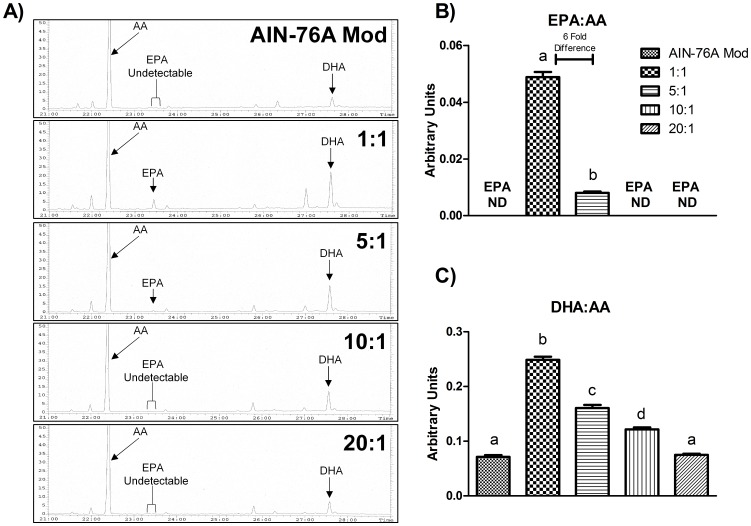
Representative (A) GC-MS chromatograms for each diet showing peaks representing AA, EPA, and DHA in AT phospholipids. (B) EPA:AA and (C) DHA:AA in AT phospholipids (n  =  10). Diets not sharing a common letter differ significantly from one another (P≤.05). ND  =  Not Detected.

**Figure 2:**
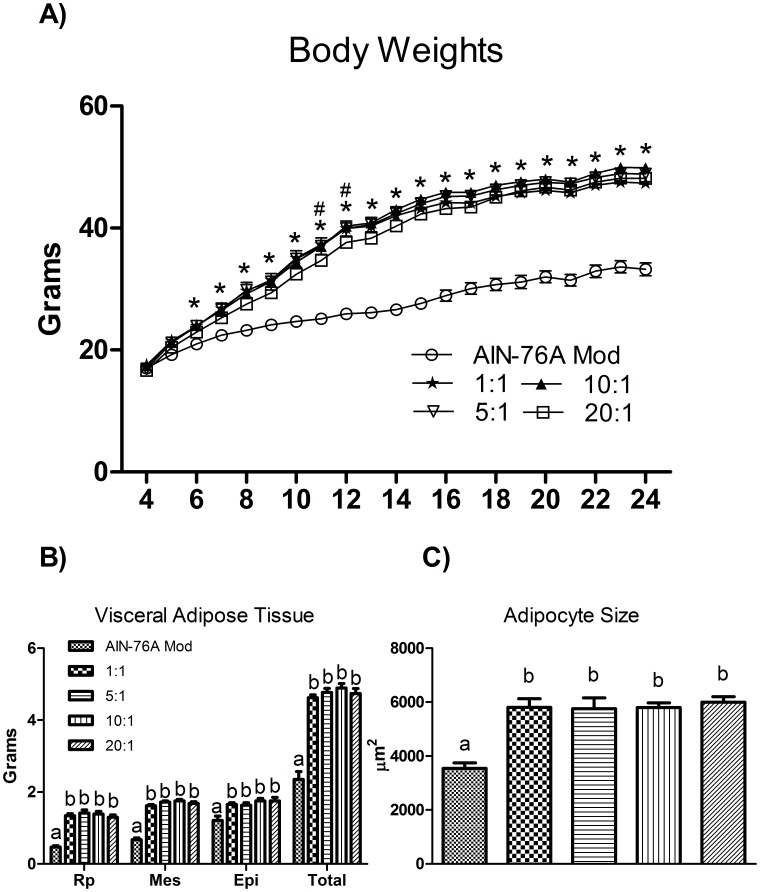
Influence of diets on (A) weekly mean body weight, (B) fat pad weights (retroperitoneal, mesentery, epididymal), and (C) adipocyte size at sacrifice (n  =  10). Diets not sharing a common letter differ significantly from one another (P≤.05). ^#^Significantly different from AIN-76A Mod (ages 6–24 weeks: all HFDs) ^#^Significantly different from 20:1 (11 weeks of age: 10:1, 12 weeks of age: 5:1) (P≤.05).
